# Sensitive and Specific Immunohistochemical Diagnosis of Human Alveolar Echinococcosis with the Monoclonal Antibody Em2G11

**DOI:** 10.1371/journal.pntd.0001877

**Published:** 2012-10-25

**Authors:** Thomas F. E. Barth, Tobias S. Herrmann, Dennis Tappe, Lorenz Stark, Beate Grüner, Klaus Buttenschoen, Andreas Hillenbrand, Markus Juchems, Doris Henne-Bruns, Petra Kern, Hanns M. Seitz, Peter Möller, Robert L. Rausch, Peter Kern, Peter Deplazes

**Affiliations:** 1 Institute of Pathology, Ulm University, Ulm, Germany; 2 Consiliary Laboratory for Echinococcosis, Institute of Hygiene and Microbiology, University of Würzburg, Würzburg, Germany; 3 Division of Infectious Diseases, University Hospital and Medical Center, Ulm, Germany; 4 Department of Surgery, Division of General Surgery, University of Alberta, Edmonton, Alberta, Canada; 5 Department of General, Visceral, and Transplantation Surgery, University Hospital of Ulm, Ulm, Germany; 6 Department of Diagnostic and Interventional Radiology, University of Ulm, Ulm, Germany; 7 Institute of Epidemiology and Medical Biometry, Ulm University, Ulm, Germany; 8 Institute of Medical Parasitology, University of Bonn, Bonn, Germany; 9 Department of Comparative Medicine, School of Medicine, University of Washington, Seattle, Washington, United States of America; 10 Institute of Parasitology, University of Zurich, Zurich, Switzerland; Universidad Peruana Cayetano Heredia, Peru

## Abstract

**Background:**

Alveolar echinococcosis (AE) is caused by the metacestode stage of *Echinococcus multilocularis*. Differential diagnosis with cystic echinococcosis (CE) caused by *E. granulosus* and AE is challenging. We aimed at improving diagnosis of AE on paraffin sections of infected human tissue by immunohistochemical testing of a specific antibody.

**Methodology/Principal Findings:**

We have analysed 96 paraffin archived specimens, including 6 cutting needle biopsies and 3 fine needle aspirates, from patients with suspected AE or CE with the monoclonal antibody (mAb) Em2G11 specific for the Em2 antigen of *E. multilocularis* metacestodes. In human tissue, staining with mAb Em2G11 is highly specific for *E. multilocularis* metacestodes while no staining is detected in CE lesions. In addition, the antibody detects small particles of *E. multilocularis* (spems) of less than 1 µm outside the main lesion in necrotic tissue, liver sinusoids and lymphatic tissue most probably caused by shedding of parasitic material. The conventional histological diagnosis based on haematoxylin and eosin and PAS stainings were in accordance with the immunohistological diagnosis using mAb Em2G11 in 90 of 96 samples. In 6 samples conventional subtype diagnosis of echinococcosis had to be adjusted when revised by immunohistology with mAb Em2G11.

**Conclusions/Significance:**

Immunohistochemistry with the mAb Em2G11 is a new, highly specific and sensitive diagnostic tool for AE. The staining of small particles of *E. multilocularis* (spems) outside the main lesion including immunocompetent tissue, such as lymph nodes, suggests a systemic effect on the host.

## Author Summary

Echinococcosis is a life-threatening disease in humans that is caused by the larval stages of the tapeworms *Echinococcus multilocularis* and *Echinococcus granulosus*. The eggs of the parasites are released with faeces of canids, and humans are aberrantly infected. In humans, the larval stages of the parasites cause tumour-like lesions mainly in the liver and the lungs. Precise diagnosis of the parasite responsible for human disease is of utmost importance since therapy regimens largely differ between cystic and alveolar echinococcosis. Diagnosis is based on serology, imaging and histology, the latter being the gold standard. However, conventional histology cannot always clearly identify the causative parasite because both parasites can cause human tissue to present similar features. Therefore, we have developed the monoclonal antibody Em2G11 and an immunohistological technique that allows a cheap and fast clear-cut diagnosis of *E. multilocularis* even on aspirates and small archived bioptic tissue samples. Furthermore, this technique disclosed an unknown feature of human alveolar echinococosis we called "small particles of *E. multilocularis*" (spems). We argue that these small particles represent micro-fragments of *E. multilocularis* and thus point to a new form of host-parasite interaction.

## Introduction

Echinococcosis is a zoonosis caused by larval stages (metacestodes) of tapeworms of the genus *Echinococcus*. In humans, alveolar echinococcosis (AE), provoked by *E. multilocularis*, and cystic echinococcosis (CE), induced by *E. granulosus sensu lato* (a complex of several genotypes or species), are particularly important since these two forms have a wide geographic distribution and may cause life threatening disease [1].

*E. multilocularis* is detected in the northern hemisphere including North America, Central and Eastern Europe, northern Asia stretching to the Far East including Japan and China [2]. Several recent reports suggest that AE is emerging. In Switzerland, for example, the incidence 2001–2005 increased by more than twofold compared to previous years (1993–2000). Several reports have also highlighted increasing numbers of cases in the Baltic countries (e.g. Lithuania) and Asia [3], and AE has also spread to the Japanese island of Hokkaido in the last decades [4]. *E. granulosus* has a cosmopolitan distribution including South and East Europe, the Middle East, Africa, Asia, North and South America. Several regions endemic for AE as well as CE have been recognised in Kirgizstan and north-western, central and north-eastern China.

The heteroxenic life cycles of both parasites are characterized by two mammalian hosts. The adult stages live in the intestine of carnivores (definitive hosts), mainly foxes and other wild canids and dogs for *E. multilocularis*, and predominantly dogs for *E. granulosus* [1]. Eggs are released into the environment with carnivore faeces. Upon uptake of eggs containing an embryonic stage (oncosphere) by intermediate hosts such as wild and domestic herbivores and omnivores, the oncospheres penetrate the intestinal mucosa and invade the portal venous system or the lymphatic system. In the capillary bed of the target organ (mainly liver and lung), the oncospheres further develop to a larval stage called metacestode that slowly grows to form a tumor-like parasitic tissue mass *(E. multilocularis)* or a cyst like structure *(E. granulosus)*. The metacestode consists of a germinal layer surrounded by a laminated layer. In natural intermediate hosts, protoscoleces with characteristic birefringent hooklets in polarisation microscopy arise within brood capsules which bud from the germinal layer [5]. In humans who are accidentally infected with parasite eggs as aberrant hosts, the pathogenicity of echinococcoses is determined by the growth capacity of metacestodes; particularly in AE this is coupled with potential metastatic dissemination. For AE, these concepts are reflected by the current WHO classification [6].

The main macroscopic difference between AE and CE is caused by different growth patterns of the metacestode. The lesions caused by *E. multilocularis* are characterized by multi-chambered (multilocular) cystic structures with root-like formations of vesicles extending to the surrounding host tissue as confirmed by digital remodelling [7]. These structures are accompanied by heavy inflammation and necrosis containing fragments of the laminated layer and particles of protoscoleces [5]. The histological hallmark of the lesion is the laminated layer synthesized by the cells of the germinal layer [8]; this laminated layer has a slender structure [5]. In contrast, the macroscopic lesion of CE is less complex and consists of large cysts of up to several centimetres, optionally containing small daughter cysts of various millimetres filled with a clear fluid. Morphologically, CE is characterized by a host-derived fibrotic capsule that surrounds the mostly unilocular cyst consisting of thick fragments up to 5 mm of the strongly periodic acid-Schiff (PAS) positive laminated layer. Inflammation is less pronounced [5], [9].

Definitive diagnosis of AE is of utmost importance since prognosis and treatment differs fundamentally from CE [10]. In all patients with AE, benzimidazoles are mandatory temporarily after complete resection of the lesions, and for life in all other cases [10]. For CE, in contrast, depending on the stage of the disease, watch and wait, drug treatment with benzimidazoles, percutaneous treatment or surgery with complete cyst removement are recommended [10]. Diagnosis of infection in humans is based on the identification of infiltrative or cystic lesions by imaging techniques such as ultrasonography or computed tomography [10]. For AE, the diagnosis is strengthened by immunodiagnostic tests, i. e. enzyme-linked immunosorbent assays (ELISAs) using native protoscolex or metacestode antigens, purified fractions (Em2 antigen), or recombinant antigens (II/3-10-, Em10- or Em18-antigen) with variable sensitivities and specificities [10]–[12]. Molecular diagnostic tools, such as polymerase chain reaction (PCR), have been used increasingly to confirm the echinococcal aetiology of lesions, also in unusual locations.

Diverse protocols have been developed and PCR is accepted as a complementary diagnostic tool for echinococcosis [13].

In humans, the histological detection of the laminated layer is crucial since protoscoleces and hooklets are very rarely seen. The laminated layer of both, *E. multilocularis and E. granulosus* metacestodes, mainly consists of polysaccharide protein complexes with a predominance of galactosamine over glucosamine [14]. The high amount of polysaccharides in the laminated layer is responsible for the high affinity to PAS staining in both species [15]. The mucin-type Em2 antigen in the laminated layer of the *E. multilocularis* metacestodes escapes the host immune response in animal models [16], [17] by modulating the T cell response and activating a T-cell-independent B-cell reaction which lacks antibody maturation [18]–[20]. Therefore, the Em2 antigen might have a pivotal role in parasite-host interaction also in humans.

The present study validated the immunohistochemical diagnoses of AE using the monoclonal antibody mAb Em2G11 on a large number of paraffin embedded samples from resection specimens and from cutting needle biopsies and fine needle aspirates of patients with histologically confirmed or with putative diagnosis of AE or CE.

## Materials and Methods

### Patients, tissue samples and aspirates

Paraffin blocks of 96 patients were available from the archives of the Institute of Pathology, University of Ulm and dated back until 1989. In compliance with the German law for correct usage of archival tissue for clinical research [21] the blocks were anonymized. The specimens were resection samples in 87, cutting needle biopsies in 6 and aspiration material in 3 cases. The patients' characteristics are given in Table 1.

**Table 1:** Patients' characteristics and localization of probe.

	AE	CE	Σ
Patients (n)	49 (51%)	47 (49%)	96
Male	21	26	47
Female	28	21	49
Males, mean age (range)/median	49.0 (18–77)/48	38.1 (13–66)/39	43.0 (13–77)/43
Females, mean age (range)/median	51.8 (18–78)/57	41.9 (11–73)/41	47.6 (11–78)/49
Overall mean age (range)/median	51.0 (18–78)/53	39.8 (11–73)/40	45.3 (11–78)/45
Patients' origin			
- Germany	47	18	65 (68%)
- Eastern Europe and Balkan peninsula	2	29	31 (32%)
Resection specimen	42	45	87
- Liver	37 (incl. 2 regional lymph nodes)	28	65
- Retropancreatic lymph node	1	-	1
- Lung	1	5	6
- Bone	1	4	5
- Omentum majus	1	1	2
- Retroperitoneum	1	-	1
- Muscle (one disseminated in lower extremity)	-	3	3
- Paravertebral	-	1	1
- Region not known	-	2	2
- Disseminated manifestation in lung and heart	-	1	1
Cutting needle biopsy from liver lesions	6	-	6
Aspiration cytology liver*/large bile duct§/gluteus maximus'	1*	1§ and1′	3

### Staining procedures

Haematoxylin and eosin (H&E) and Periodic acid-Schiff (PAS) stainings were performed according to standard protocols.

### Immunohistochemistry/Immunocytology

MAb Em2G11 is an *in vitro* produced monoclonal IgG_1_ antibody from a mouse hybridoma cell line as described in detail elsewhere [16]. The antibody is available on request from P. Deplazes at the Institute of Parasitology, University of Zurich, Switzerland. For immunohistochemistry, standard protocols were used [22]. Briefly, for antigen retrieval, the sections were heated in citrate buffer at pH 6 in a microwave oven for 20 minutes. The primary antibody was used in a concentration of 0.2057 mg/ml in phosphate buffer saline (PBS); slides were incubated with 50 µl per section in a humid chamber at room temperature for 30 minutes. As detection system we used the EnVision Kit (Dako, Carpintera, CA, USA) according to the manufacturer's protocols.

These samples were first analyzed by two of us (TFEB and TSH) on a multihead microscope using slides stained with H&E and PAS. A consensus diagnose was achieved for each sample. Baseline criteria for diagnosis on histological grounds were as follows (AE *versus* CE): shape of the laminated layer (slender *versus* broad); histological growth pattern (a tubular growth pattern with ill defined borders *versus* pseudocystic and a well demarked lesion with a fibrous capsule); and the presence *versus* the absence of necrosis; see Table 2).

**Table 2:** Conventional macroscopic and histological diagnostic criteria for the differential diagnosis of AE and CE [5].

	AE	CE
Macroscopic view	Multiple cysts	Solitary cyst
Histological view		
- Laminated layer	Slender (<1 mm)	Thick (up to 3 mm)
- Necrosis	Abundant	Scarce
- Growth pattern	Tubular	Pseudocystic
- Encapsulation by host	Ill deliminated	Fibrous capsule

As controls for the immunohistochemistry, we included tissue from archived paraffin samples with caseous necrosis of tuberculosis (n = 2), fibrinoid necrosis from rheumatoid nodule (n = 2), and cases with areas of micro necrosis from sarcoidosis (n = 2) as wells as samples each from necrotizing osteoblastic osteosarcoma (n = 2) and colon carcinoma (n = 2).

Furthermore, sections of paraffin embedded tissue from a Mongolian jird *(Meriones unguiculatus)* experimentally infected with *E. multilocularis* were stained. For this, homogenized parasitic tissue grown in culture was injected intraperitoneally and the animal was sacrificed after an abdominal swelling was observed after a few months. *E. multilocularis* tissue was recovered from the peritoneum, formalin fixed and paraffin embedded.

### Ethics statement

The animal experiments were carried out in accordance with German regulations on the protection of animals (animal protection law). Ethical approval of the study was obtained from the ethics committee of the government of Lower Franconia (621.2531.01-2/05).

## Results

### Patients

The total number of patients included was 96. According to the medical records, 47 patients with diagnosis AE originated from Germany, two were from Eastern Europe or the Balkans (Table 3). For CE, 18 patients were supposed to be German having a migrative background from Eastern Europe. 29 had an Eastern European or Balkanese background including one patient from Greece.

**Table 3:** Cases with difficult histological/cytological diagnoses.

No.	Organ	Laminated layer	Necrosis	Fibrous capsule	Growth pattern	Further peculiarities	Diagnosis with H&E + PAS stain	Diagnosis with Em2G11
**Histology**								
1	Liver tissue and gallbladder with fragments of a pseudocyst	Slender	Yes	Yes	Tubular		AE	AE
2	Left hemipelvectomy	Predominantly slender	Yes	No	Not tubular		CE	CE
3	Femur	Predominantly thick	Yes	No	Expansive growth along trabeculae		CE	CE
4	Fragments of ilium and left proximal femur	Slender	Yes	Yes	Tubular		AE	CE
5	Lung	Few, slender	Yes	No	Not tubular	Necrotizing inflammation with strong fibrotic reaction; some protoscolices	AE	CE
6	Right partial hepatectomy (segment VIII)	Intermediate	Yes	Yes	Not tubular	Calcifications	AE	CE
7	Liver, gallbladder and thoracic diaphragm	Slender	Yes	Yes	Not tubular	Calcifications	AE	CE
8	Liver and gallbladder	Slender and thick	Yes	Strong	Not tubular		CE	AE
9	Right Lung, lower lobe	Thick compressed, multilayer	Yes	Yes	Not tubular		CE	AE
**Cytology**								
10	Muscle lesion	Predominantly thick, multilayer	Yes	No	-		CE	CE
11	Liver cyst	Slender	Yes	No	-		AE	AE
12	Liver cyst	Predominantly thick, multilayer	No	No	-		CE	CE

Using the above mentioned classical histomorphological and histochemical criteria [5] summarized in Table 2, 49 (51%) of the samples were classified as AE, and 47 (49%) were diagnosed as CE. In 12 of these 96 samples histological diagnosis was difficult since the above mentioned criteria did not lead to a clear-cut diagnosis even after extensive discussions on the multihead microscope (Table 3). These samples showed partly overlapping characteristics that clouded the diagnostic criteria. The diagnostic difficulties are summarized as follows: a) tissue samples from bone lesions since in these cases, laminated layers were always slender and their structure did not allow a reliable diagnosis; b) tissue samples with extensive zones of necrosis combined with thick or intermediate laminated layers; c) only very few particles of the laminated layer; and d) no clearly identified tubular growth pattern. Three samples with echinococcosis were aspirates from a liver lesion, a muscle lesion and from the common bile duct. Cytology was regarded as challenging since on the slides only disrupted fragments of the laminated layer without context to the surrounding were present. Details of the cases are given in Table 1.

### Staining modalities of mAb Em2G11 *in situ*

**Figure 1:**
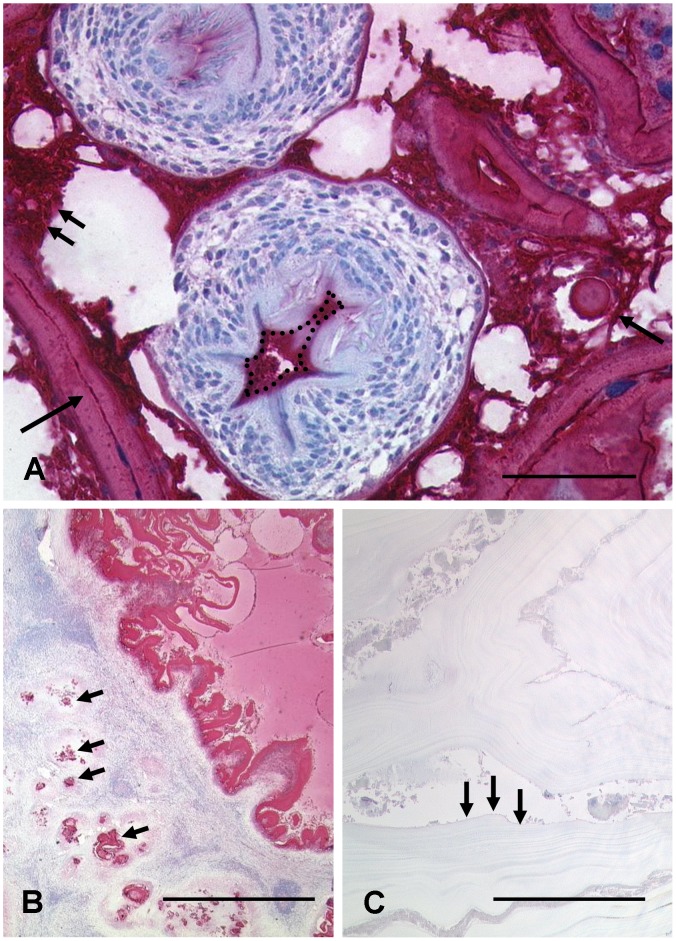
Immunohistochemical staining modalities of the monoclonal antibody (mAb) Em2G11 for metacestodes of *Echinococcus multilocularis*. Figure 1A: In metacestodes grown in a Mongolian jird, the antibody strongly marks the laminated layer (single arrow left below). The germinal layer and calcareous corpuscles are strongly stained (two arrows and single arrow right) as well as the precipitated cyst fluid. The area oft the rostellum is superimposed with a positive reacting layer (dashed line) while the inner part of the protoscolex did not react with the monoclonal antibody; bar =  50 µm. 1B, C: In human liver, the Em2 antigen is strongly positive in the slender laminated layer of *E. multilocularis*. The staining reveals a tubular and infiltrative growth pattern (arrows). In contrast, the laminated layer of *E. granulosus* is much broader (arrows), no staining is detected by mAb Em2G11; bar =  1000 µm.

We first analysed sections of a jird experimentally infected with *E. multilocularis*. Both the laminated and the germinal layers, the calcareous corpuscles as well as the cyst fluid content were strongly positive. In contrast, the protoscoleces did not react with mAb Em2G11. However, a dense layer surrounding the protoscoleces was positive (Fig. 1A).

**Figure 2:**
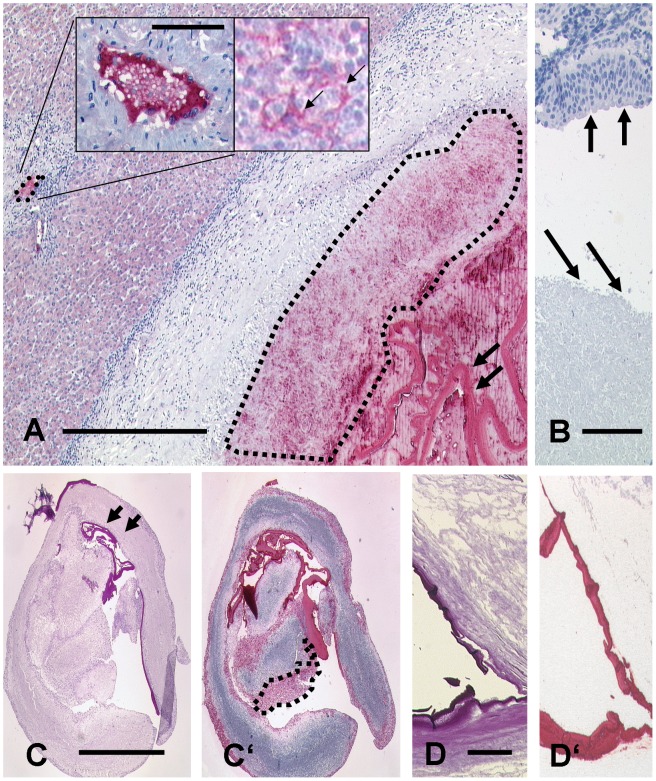
Immunohistochemical staining modalities of the monoclonal antibody (mAb) Em2G11 for metacestodes of *Echinococcus multilocularis*. Figure 2A: *E. multilocularis* lesion in human liver tissue. The antigen is detected in the laminated layer (two arrows, right) and in the necrotic area around the lesion (dashed lined area, right). The antibody detects small particles of *E. multilocularis* (spems) up two 1.5 mm away from the main lesion in a small liver vessel (small area marked with a dashed line on the left). Insert left highlights this lesion at a higher magnification showing a specific staining of spems. Insert right shows specific staining in lymphoid tissue of a regional lymph node on the surface of cells (arrows; bar =  750 µm; bar insert =  40 µm). B: In contrast, no staining is observed in caseous necrosis of tuberculosis (arrows low) and in bronchial epithelial tissue (arrows high; bar =  50 µm). C: Serial section of an aspirate from the liver. C shows a PAS staining of a strongly positive laminated layer. C′: Staining of the section with mAb Em2G11 reveals a strong positivity of the laminated layer and of the necrotic tissue with spems (dashed lined area; bar =  500 µm). D: PAS staining of brain tissue showing the laminated layer of an *E. multilocularis* metacestode. D′: The laminated layer is strongly positive for mAb Em2G11 even after 60 years of formalin fixation (bar =  50 µm).

In human AE samples we found the following staining pattern: 1. The laminated layer of *E. multilocularis* metacestodes was always strongly positive. Even small fragments were easily detected in the solid tissue samples as well as in the aspirates. In contrast, no staining at all was detected in the laminated layer of *E. granulosus* (Fig. 1B, C). 2. The necrotic areas surrounding the laminated layer of *E. multilocularis* showed a fine granular staining. This positive granular staining was also visible up to 1.5 millimetres away from the main lesion in sinusoids or lymphoid tissue of adjacent liver tissue surrounding the metacestode and even in two regional lymph nodes of hepatic AE (Fig. 2A). This observation was also confirmed in one patient with initial cutting needle biopsy of a liver lesion that contained only little diagnostic material. By microscopy we found necrotic material with some very small PAS positive fragments suspicious for echinococcosis. Immunohistology with the mAb Em2G11 showed a strongly positive reaction of the small fragments in the necrotic tissue. The liver lesion was resected. Histology of the resection specimen clearly proved a full blown lesion of *E. multilocularis*. The same result was obtained from an aspiration fluid from a liver lesion of a 73-year-old woman. Cytology revealed some slender fragments of a laminated layer and necrotic tissue. Immunocytology showed a strong reaction of the laminated layer and of the necrotic tissue, confirming the diagnosis of AE (Fig. 2C). The two other aspirates were clearly negative for staining with mAb Em2G11 leading to the diagnosis of CE. Therefore, immunohisto/cytochemistry with mAb Em2G11 is a reliable method also on very small samples for definitive diagnosis of AE.

To rule out that staining was an unspecific reaction of necrotic tissue, we stained a series of necrotic lesions including caseous necrosis from pulmonary tuberculosis, fibrinoid necrosis from nodules of patients with rheumatoid arthritis, samples with sarcoidosis and tumor samples of high-grade osteoblastic osteosarcoma, adenocarcinoma of the lung and invasive colon carcinoma with areas of tumour necrosis (Fig. 2B). Furthermore, a metacestode of *Taenia solium* resected from a brain lesion of young woman returning from a journey in Nepal was stained. No traces of mAb Em2G11-related staining patterns were observed in any of these controls. No unspecific staining was observed when the primary antibody was omitted and even in liver tissue with known high endogenous peroxidase activity we did not notice any unspecific staining with this detection system.

To test the efficiency of the antibody on long term formalin conserved tissue we were able to include a sample of an AE case described by Rausch and Schiller in 1956. The tissue samples were from a 28-year-old male Inuit who was symptomatic with headache and disturbance of vision in 1950 in Alaska. Craniotomy had been performed and “a mass of increased resistance” had been removed from his brain [23] and specimens measuring up to 6 mm had been kept in formalin for 61 years. This tissue was processed according to our standard techniques. Histologically, the section showed brain tissue with necrosis including a slender fragment of a PAS positive laminated layer that was strongly positive after staining with the mAb Em2G11. We conclude that the antigen detected by mAb Em2G11 is highly stable over time in formalin fixed tissue (Fig. 2D).

We next revised the specimens of 12 cases which had been considered difficult to diagnose by applying the classical criteria (Table 2) on conventional histology and cytology (H&E and PAS staining; Table 3). In 3 out of the 12 cases, immunohistochemistry confirmed the diagnosis made by conventional histology (AE, ×1; CE, ×2). However, the diagnosis had to be corrected for 6 patients (AE to CE, ×4; CE to AE, ×2). The three aspirates were clearly identified as AE (×1) or CE (×2).

Therefore, misdiagnosis was encountered in 6 cases with conventional histology (sensitivity: 0.957; specificity: 0.918).. The diagnostic problems are as follows. *E. granulosus* metacestodes were growing in bone and the laminated layer was extraordinarily small and slender thus mimicking features of AE (no. 4). Even specimens taken from lung, liver, or gallbladder can consist of rather thin or fragments of the laminated layers in necrotic tissue and lead astray to AE despite *E. granulosus* is the correct metacestode (no. 5, 6, 7). In contrast, a broad fibrotic capsule and fragments of a thick laminated layer can be observed in AE and feign CE (no. 8). The specimen of case no. 9 showed a compressed thick laminated layer without tubular growth pattern and CE was erroneously considered.

In the three samples of aspiration cytology, the diagnoses of one lesion of AE and two lesions of CE were confirmed by immunocytochemistry.

## Discussion

Diagnosing AE and CE in humans requires the integration of clinical findings, imaging results and classical histopathology, supplemented by molecular (PCR) detection, and serology [10].

The monoclonal antibody mAb Em2G11 recognizes an epitope of a mucin-type carbohydrate antigen called Em2 [17] which is a major antigen of the laminated layer of the *E. multilocularis* metacestode that is also present in the cyst fluid [16], [20]. The laminated layer is a key factor in the parasite's survival strategy [24] and therefore is an ideal diagnostic parasite target. Fragments of this structure have even been demonstrated in “died out” lesions in humans [25] or animals [26].

We show that the mAb Em2G11 is strongly positive in the laminated layer of *E. multilocularis* lesions in various human tissues in all samples studied. Protoscoleces could not be found in the investigated material of 49 AE patients confirming that protoscoleces are a very inconstant diagnostic feature [5]. Therefore, the mAb Em2G11-positive laminated layer is the crucial immunohistological hallmark for diagnosis of AE.

Besides the positive laminated layer, we found positive signals in the necrotic zone surrounding the metacestode. Since it is well known that necrotic tissue may cause unspecific immunohistochemical staining, we evaluated this finding by staining various control tissues containing different types of necrosis. None of these controls was positive. We conclude that, in the necrotic tissue, small mAb Em2G11-positive acellular particles of less than 1 µm are present. We also detected these small fragments as positive signals in liver sinusoids up to 1.5 mm away from the defined lesion as well as in lymphoid aggregates near the main lesion. We termed these small mAb Em2G11-positive particles of *E. multilocularis* (spems). Excretory and secretory products have been described from numerous helminth parasites, with a putative role of these substances in tissue invasion and/or immunomodulation. In *E. multilocularis*, some excretory/secretory products have been further characterized, such as peptidases [27]. A recently published study has described the ability of some excretory/secretory products to induce apoptosis in dendritic cells [28]. However, in these studies, only single molecules, or a mixture of molecules, were described and analyzed, mainly in *in vitro* culture studies. We here describe, as a novelty, the visualization of corpuscles (and not soluble molecules) with the help of the monoclonal antibody.

We suggest that these *spems* are shed from the laminated layer into the surrounding tissue and may flow through the body via blood vessels and the lymphatics. The role of these *spems* remains to be elucidated. Nevertheless it can be hypothesized that parts of these acellular fragments represent remnants of the metacestode that may be involved in the complex immunological processes surrounding the parasite including apoptosis in dentritic cells [24], [28]. In line with this observation, we confirmed involvement of regional lymph nodes with fragments of the laminated layer of *E. multilocularis* in two patients with liver lesions underlining the capacity for dissemination of AE [25].

To distinguish between the immunohistological aspects of AE and CE, a large series of tissue samples of human patients infected with *E. granulosus* was stained using mAb Em2G11. There was no positivity at all of *E. granulosus* neither in the laminated layer, the germinal layer, calcareous corpuscles, nor in the protoscoleces confirming the high species specificity of this monoclonal antibody [16]. In our series, using the classical histopathological criteria for the differential diagnosis of AE and CE based on H&E and PAS staining, we have classified a series of 96 patients with echinococcosis. About 12% of the cases were challenging and diagnosis was fixed as ‘Diagnosis with H&E + PAS stain’ (see Table 3). The most difficult cases were bone infections of *E. granulosus* which all showed an exceptionally slender laminated layer blurring this otherwise important histological criterion for CE. Diagnostic differentiation was even more difficult in cases with isolated bone lesions of CE, and without evidence of any affected soft tissues which would allow diagnosis of CE due to the typical broad laminated layer. Further cases were difficult to classify since only few fragments of the laminated layer were present in the biopsy. In some cases, a tubular growth pattern *versus* a clearly limiting capsule could not be evaluated as histological diagnostic landmarks in the samples. By using immunohistology with mAb Em2G11, in 6 of these 12 samples, diagnosis of AE or CE had to be adjusted (6 of 96 cases *i. e.* 6.25% of all samples with AE or CE). These findings underline the high diagnostic value of mAb Em2G11 for the histological differential diagnosis of AE in situations with no clear cut histological criteria.

This immunochemical approach allowed diagnosis also on aspirates of cystic fluids from liver lesions. The precipitated parasitic material in the fluid was stained on paraffin section marking the laminated layer as well as the granular fragments within the necrotic area (Fig. 2C). Therefore, diagnostic specificity can also be achieved by immunocytology on aspiration material. In addition, we had the opportunity to test mAb Em2G11 on tissue from one of the first descriptions of *E. multilocularis* outside Europe in humans. The brain tissue was from an Alaskan Inuit and had been fixed in formalin for 60 years. Staining with the antibody on a paraffin section from this tissue showed a strong reaction with mAb Em2G11. This confirms the first description of AE in America and proves that the antigen is highly stable for long periods in formalin. This finding may be useful for exact classification of AE in humans on large scale of archived tissue regarding the distribution of *E. multilocularis* outside the yet known and defined areas with high incidence.

Radical resection is the primary goal of therapy. In the current WHO classification, the distance of the *E. multilocularis* lesion to the resection margins is proposed to be >2 cm [10]. This distance takes into account that the larva follows a diffuse, tubular growth pattern that is difficult to recognize, even histologically. Therefore, the finding of spems in the surrounding tissue may be of importance for a critical re-evaluation of the definition of surgical resection limits as one of the most important prognostic parameters.

In conclusion, our findings prove that mAb Em2G11 is a highly specific and sensitive new and easily applicable tool for specific diagnosis of AE on paraffin archived tissue. The detected small particles outside the main lesion extend knowledge of immunopathology of the parasite in humans and point to novel characteristics of the host-parasite interaction.
